# Supplementary material for: Longitudinal proteomic profiling of dialysis patients with COVID-19 reveals markers of severity and predictors of death
Source: eLife. 2021 Mar 11;10:e64827. doi: 10.7554/eLife.64827 (PMC8064756; doi:10.7554/eLife.64827)
Supplement: Transparent reporting form [file elife-64827-transrepform.docx]

***eLife’s* transparent reporting form**

We encourage authors to provide detailed information *within their submission* to facilitate the interpretation and replication of experiments. Authors can upload supporting documentation to indicate the use of appropriate reporting guidelines for health-related research (see [EQUATOR Network](http://www.equator-network.org/%20)), life science research (see the [BioSharing Information Resource](https://biosharing.org/)), or the [ARRIVE guidelines](http://www.plosbiology.org/article/info:doi/10.1371/journal.pbio.1000412) for reporting work involving animal research. Where applicable, authors should refer to any relevant reporting standards documents in this form.

If you have any questions, please consult our Journal Policies and/or contact us: [editorial@elifesciences.org](mailto:editorial@elifesciences.org).

**Sample-size estimation**

- You should state whether an appropriate sample size was computed when the study was being designed
- You should state the statistical method of sample size computation and any required assumptions
- If no explicit power analysis was used, you should describe how you decided what sample (replicate) size (number) to use

Please outline where this information can be found within the submission (e.g., sections or figure legends), or explain why this information doesn’t apply to your submission:

No formal statistical power calculations were undertaken to determine the sample size. Sample collection was performed during the first ‘wave’ of the COVID-19 pandemic in Spring 2020. Due to the formidable logistical challenges of collecting research samples from COVID-19 patients during a period of exceptional pressure on health services and with restrictions on laboratory staffing due to a UK national lockdown and social distancing requirements, we simply collected as many samples as possible within the limits of the clinical and research staffing that was available.

**Replicates**

- You should report how often each experiment was performed
- You should include a definition of biological versus technical replication
- The data obtained should be provided and sufficient information should be provided to indicate the number of independent biological and/or technical replicates
- If you encountered any outliers, you should describe how these were handled
- Criteria for exclusion/inclusion of data should be clearly stated
- High-throughput sequence data should be uploaded before submission, with a private link for reviewers provided (these are available from both GEO and ArrayExpress)

Please outline where this information can be found within the submission (e.g., sections or figure legends), or explain why this information doesn’t apply to your submission:

The study was an observational study using patient samples, so no experiments were performed.

Some patients had longitudinal samples taken over the course of their illness (Fig. 1b). The number of samples and the number patients for each analysis are indicated in the appropriate Figure legends and Methods.

Raw sample-level proteomic data and clinical annotation is provided as Source Data Files 1-4. In addition, these data have been deposited in the Dryad Digital Repository (doi:10.5061/dryad.6t1g1jwxj).

Sample exclusions: 3 samples failed quality control measures and were excluded. These samples failed internal control checks that are part of the Olink assay system and in addition were clear outliers on principal components analysis. In addition, 5 samples failed quality control on a single proteomic panel only, with the remaining panels passing QC. For these samples, proteins on the panel that failed QC were set to missing, and the data for the remaining proteins was retained. This is described in the Methods.

**Statistical reporting**

- Statistical analysis methods should be described and justified
- Raw data should be presented in figures whenever informative to do so (typically when N per group is less than 10)
- For each experiment, you should identify the statistical tests used, exact values of N, definitions of center, methods of multiple test correction, and dispersion and precision measures (e.g., mean, median, SD, SEM, confidence intervals; and, for the major substantive results, a measure of effect size (e.g., Pearson's r, Cohen's d)
- Report exact p-values wherever possible alongside the summary statistics and 95% confidence intervals. These should be reported for all key questions and not only when the p-value is less than 0.05.

Please outline where this information can be found within the submission (e.g., sections or figure legends), or explain why this information doesn’t apply to your submission:

Information on statistical analysis is described within the figure legends and methods. Due to the high-dimensional nature of the data (large sample sizes and large numbers of proteins measured), displaying raw data was not possible. However, the underlying raw data are made available as Source Data Files 1-4. Exact P-values before and after multiple testing correction are reported for all proteins in all statistical analyses (Supplementary File 1 c, d, f, g, h).

(For large datasets, or papers with a very large number of statistical tests, you may upload a single table file with tests, Ns, etc., with reference to sections in the manuscript.)

**Group allocation**

- Indicate how samples were allocated into experimental groups (in the case of clinical studies, please specify allocation to treatment method); if randomization was used, please also state if restricted randomization was applied
- Indicate if masking was used during group allocation, data collection and/or data analysis

Please outline where this information can be found within the submission (e.g., sections or figure legends), or explain why this information doesn’t apply to your submission:

No experiments were done as this was an observational study.

Plate layouts was designed through a combination of ensuring case/control balance across plates with random selection of samples from each category and random ordering of allocation to wells (described in the Methods).

**Additional data files (“source data”)**

- We encourage you to upload relevant additional data files, such as numerical data that are represented as a graph in a figure, or as a summary table
- Where provided, these should be in the most useful format, and they can be uploaded as “Source data” files linked to a main figure or table
- Include model definition files including the full list of parameters used
- Include code used for data analysis (e.g., R, MatLab)
- Avoid stating that data files are “available upon request”

Please indicate the figures or tables for which source data files have been provided:

The data underlying all analyses (raw sample-level proteomic data and clinical annotation) are provided as Source Data Files 1-4. In addition, these data have been deposited in the Dryad Digital Repository (doi:10.5061/dryad.6t1g1jwxj).

**Code availability:** code is available in the following GitHub repository: https://github.com/jackgisby/longitudinal_olink_proteomics

In addition:

-The summary statistics in Fig. 3a are contained in Supplementary File 1c.

-The summary statistics in Fig. 3b are contained in Supplementary File 1d.

-The raw data underlying the heatmap in Fig. 3c are contained in Source Data Files 1-2.

-The raw data underlying the violin plots in Figures 5 and 6 are contained in Source Data Files 1-2.

-The data underlying Fig. 7a are contained in Supplementary File 1e.

-The data underlying Fig. 7b are contained in Supplementary File 1f.

- The summary statistics in Fig. 8 are contained in Supplementary File 1g.

-The raw data underlying Fig. 9 are contained in Source Data Files 1-2 and the summary statistics are contained in Supplementary File 1h. The code used to generate the Figures is available in the GitHub link above.
